# Risk-Adapted Use of Vancomycin in Secondary Scoliosis Surgery May Normalize SSI Risk in Surgical Correction of High-Risk Patients †

**DOI:** 10.3390/jpm14101017

**Published:** 2024-09-24

**Authors:** Nima Taheri, Paul Köhli, Zhao Li, Zhen Wang, Tu-Lan Vu-Han, Konstantin Cloeren, Antonia Koch, Serafeim Tsitsilonis, Friederike Schömig, Thilo Khakzad, Matthias Pumberger

**Affiliations:** 1 Center for Musculoskeletal Surgery, Charité—Universitätsmedizin Berlin, Corporate Member of Freie Universität Berlin, Humboldt-Universität zu Berlin, 10117 Berlin, Germany; 2 Berlin Institute of Health, Julius Wolff Institute for Biomechanics and Musculoskeletal Regeneration, Charité—Universitätsmedizin Berlin, Augustenburger Pl. 1, 13353 Berlin, Germany; 3 BIH Charité Junior Clinician Scientist Program, Berlin Institute of Health at Charité—Universitätsmedizin Berlin, BIH Biomedical Innovation Academy, 10117 Berlin, Germany

**Keywords:** secondary scoliosis, vancomycin, surgical site infection, pediatric surgery

## Abstract

**Introduction:** Intrawound application of vancomycin is becoming increasingly controversial for the prevention of surgical site infection (SSI). As children undergoing spinal fusion for secondary scoliosis are at high risk for SSIs, evidence regarding the impact of intraoperative vancomycin installation on SSI rates in these patients is of utmost importance. **Methodology:** A single surgeon cohort of patients under 18 years of age undergoing surgery for secondary scoliosis in 2017 was analyzed with regard to the development of SSIs requiring surgical revision and adverse events. Use of vancomycin was restricted to cases with higher risk of infection. Patients undergoing distraction surgery for growing devices were excluded. **Results:** After exclusions, 64 patients remained (vancomycin n = 39, control n = 25). The SSI rates were 12.8% in patients receiving vancomycin (n = 5/39) and 4% in the control group (n = 1/25, *p* = 0.785). None of the patients suffered from adverse events. Univariable logistic regression revealed younger age (*p* = 0.03) and meningomyelocele as predictors for SSI (*p* = 0.006), while the high-risk group receiving vancomycin was not at higher odds for SSI, also after adjustment for possible confounders such as age or MMC (*p* = 0.031; *p* = 0.009). **Discussion:** SSI rates were comparable between groups, suggesting a normalization of SSI risk in the vancomycin-treated patients with a preoperatively increased risk of SSI. Future, larger studies in these rare diseases are needed to confirm these results.

## 1. Introduction

Secondary scoliosis is defined as a progressive lateral curvature due to either neurological or muscular diseases or embryological malformation of one or more vertebrae. It often requires surgical treatment to provide spine and thoracic growth and to allow patients to maintain good lung function and the ability to sit or walk [1,2,3]. Despite the necessity of surgery to improve the overall outcome and quality of life in these patients, the complication rate is high, especially for surgical site infections (SSIs) [4]. The general infection rate after deformity surgery regardless of the underlying etiology has been shown to be as high as 2.8 to 9.2% [4,5,6,7,8]. For posterior spinal fusion in secondary scoliosis, rates between 6 and 24% have been reported [9]. The consequences are serious: prolonged antibiosis, surgical debridement, or in some cases implant removal and exchange are necessary. SSI treatment on average costs USD 20.785 per patient in the USA [10].

Recent research has evaluated various antibiotic protocols, povidone scrubs, as well as the supra- or subfascial intrawound application of vancomycin in pediatric spine surgery to minimize SSI rates [11,12,13]. While Armaghani was able to prove serum levels of vancomycin to be nontoxic in 228 cases of posterior spinal fusion (PSF) in a combined cohort of both adolescent idiopathic and secondary scoliosis as well as spondylolisthesis and trauma [13], contradictory results have been published regarding the effect on SSI incidence. In their respective studies, both Mallet et al. and Garg et al. found that vancomycin does not have an influence on SSI incidence in cohorts of children with adolescent idiopathic scoliosis undergoing PSF [11,14]. In contrast, Thompson et al. observed a significant reduction in SSI incidence after the use of vancomycin. Vancomycin also reduced SSI incidence after prior surgeries without application of vancomycin in their retrospective cohort study [15].

In addition, there is growing concern regarding microbial selection pressure after vancomycin application both in a pediatric and in an adult population.

Gans et al. showed an increase in Enterobacter and methicillin-resistant Staphylococcus aureus (MRSA) in a pediatric population [16], while other groups reported more Gram-negative infections in the treated groups [17,18].

As the application of vancomycin has no international consensus in pediatric spinal deformity surgery, the main objective of this study was to examine the effect of intraoperative vancomycin application on the incidence of SSI, vancomycin-related adverse events, as well as changes in pathogen spectrum in cases of SSI. We aim to provide information regarding the abovementioned parameters in a patient collective at high risk of SSI undergoing PSF compared to patients for whom an active decision was made to not use vancomycin due to lower risk. We hypothesize that intraoperative vancomycin use is associated with a normalization of the incidence of SSI after posterior spinal fusion (PSF) or implantation of a distractible transpedicular instrumentation in children with secondary scoliosis.

## 2. Material and Methods

### 2.1. Study Collective

The study was approved by the institutional ethics committee (project number: EA 2/220/21). Patients undergoing posterior spinal instrumentation for secondary scoliosis in our orthopedic department between 2017 and 2021, who were operated on by the head of deformity surgery, were identified from discharge letters and diagnostic procedural codes and were included retrospectively. Patient data were handled and analyzed within the hospital’s secured clinical IT infrastructure by authorized study personnel only.

All patients undergoing PSF or implantation of a distractible transpedicular instrumentation for secondary scoliosis younger than 18 years and operated on between 01/2017 and 10/2021 were included in our study. Patients who were older than 18 years or undergoing distraction of a growing rod system were systematically excluded. Patients with apparent infection, history of Stevens–Johnson syndrome, Red man syndrome, or vancomycin allergy were also excluded. Patients were retrospectively split into two groups, with the interventional group having subfascial intrawound application of 2 g vancomycin and the control group/no-Vanco group not receiving vancomycin, depending on the surgeon’s judgment of the individual case’s SSI risk.

### 2.2. Perioperative and Surgical Treatment with/without Intrawound Vancomycin Application

Surgical procedures were performed by a single spine surgeon and were strictly PSF, implantations of a distractible construct with a pedicle screw–rod construct, or the conversion of a distractible construct to final PSF. Thirty minutes before skin incision, preoperative antibiosis in the form of Cefazolin was applied intravenously using 30–40 mg per kilogram of body weight and was repeated if surgery took longer than 3 h up until to a maximum dosage of 100 mg per kilogram body weight per 24 h. In case of penicillin allergy, intravenous vancomycin (30–40 mg per kilogram of body weight) was applied as preoperative antibiosis. After induction of general anesthesia and orotracheal intubation, patients were placed on the operating table in prone position. The surgical area was first cleaned with propanol disinfection using SoftaseptN (Fa. Braun). This was followed by four rounds of disinfection with an octenidindihydrochlorid/propanol solution (Octeniderm, Fa. Schülke & Mayr) for a minimum of 3 min. After disinfection, sterile surgical drapes were applied, and fixation was ensured with an incision foil (Ioban, Fa. 3 M). Before wound closure, wound irrigation with at least 4 L of ringer lactate was performed. Depending on the surgeon’s assessment, 2 g of vancomycin were added subfascially within the surgical site and evenly distributed using forceps. The factors influencing this decision were surgery duration, invasiveness, patient condition and concerns regarding intraoperative sterility, but these were subjective in nature. After vancomycin application, the surgical incision was then closed in layers, ensuring a water-tight closure of the fascia, followed by subcutaneous and cutaneous sutures. The wound was covered with dry, sterile compresses, which were secured in place with Fixomull Dressing Tape (BSN Medical). The first routine postoperative dressing change was performed on the 2nd postoperative day, followed by daily changes of the sterile drapes.

### 2.3. Data Collection

Patient data, surgical treatment and postoperative complications were extracted from physical and electronic patient records. Demographic data such as age, sex, body weight, height, and BMI, as well as preoperative comorbidities were examined and noted through analysis of medical charts and discharge data. Information about the surgical procedures (e.g., length of instrumentation, number of osteotomies, duration of surgery, possible duration of intensive care unit (ICU) stay, and the hospital stay in general were also assessed.

The primary outcome was the occurrence of SSI requiring surgical revision, while occurrences of adverse events (anaphylaxis, renal failure) were analyzed as secondary outcomes. Changes in postoperative serum creatinine levels as well as diuresis and for anaphylaxis analyzed via perioperative cardiovascular monitoring and clinical examination of the patients’ skin were indications for adverse events.

Diagnosis of SSI for indication of surgery was dependent on clinical wound examinations for signs of infection (e.g., prolonged secretion, rubor, dolor, hyperthermia, systemic symptoms) by the attending surgeon as well as laboratory changes (leukocytes, postoperative C-reactive protein (CRP), and postoperative procalcitonin (PCT)).

### 2.4. Statistical Analysis

Descriptive and comparative statistical analyses were performed via SPSS (Version 28.0.1.0). Descriptive analysis was performed using arithmetic mean, standard deviation and relative proportions. Data were analyzed for normal distribution using the Shapiro–Wilk test and in case of normal distribution, a two-sided *t*-test was performed. If normal distribution did not apply, and for ordinally scaled data, the Mann–Whitney U Test was used for further analysis. For nominal data, Fisher’s exact test was performed. Univariate and bivariate logistic regression analysis were performed in R Studio, (RStudio 2023.06.2 + 561, Posit Software, Boston, MA, USA), with the occurrence of SSI as the dependent (outcome) variable and potential risk factors and adjusted confounders as independent (predictor) variables. Potential confounders were identified based on an entry level of *p* < 0.1 in univariable regression, clinical expertise, literature review, and directed acyclic graphs created using the ‘daggity’ software (v3.1) [19]. To avoid overfitting of the models, the number of confounders adjusted for in the same logarithmic model was limited to bivariate analysis, based on the event count [20,21]. *p*-values were defined as statistically significant if they were below 0.05.

## 3. Results

### 3.1. Patient Collective

**Figure 1:**
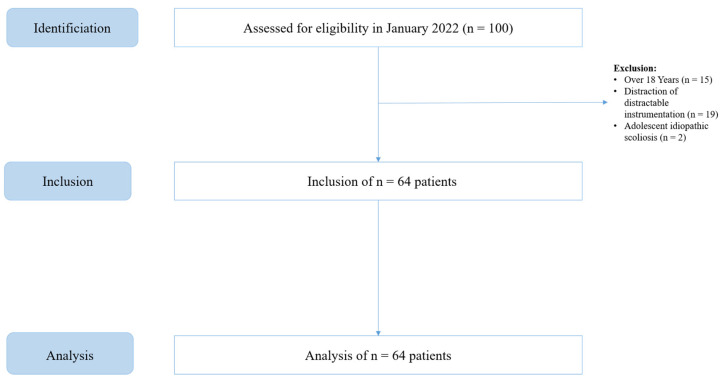
STROBE flow chart of patient in- and exclusion.

We identified 100 patients with secondary scoliosis undergoing spinal surgery. After exclusion of patients older than 18 years and those undergoing distraction surgeries for distractible constructs, 64 total patients were identified (Figure 1). Of these 64 patients, 39 patients received intraoperative vancomycin and 25 patients did not.

### 3.2. Demographic Data

There were no significant differences regarding the cohorts’ characteristics (see Table 1).

The average age was 12.6 ± 3.0 years, and 35 (55%) participants were female with an average preoperative BMI of 17.8 ± 4.8 and a height of 1.42 m ± 0.18 with no significant differences between the groups. Regarding the distribution of underlying syndrome, ASA score and the number of preoperative spinal surgeries, no significant differences were observed.

### 3.3. Surgical and Anesthesiology Data

Both surgery (177.6± 55.8 vs. 184.96± 65.4 min), *p* = 0.633) and anesthesia were comparable in both groups (334.5 ±70.5 vs. 352.8 ± 77.8 min, *p* = 0.333).

There were no significant differences regarding the type of surgery (*p* = 0.784) or length of instrumentation (13.3 ± 1.8 vs. 12.7 ± 2.8 segments, *p* = 0.498). There were no significant differences regarding duration of ICU (2.7 ± 3.1 vs. 2.4± 3.1 days; *p* = 0.4974; Table 2). and hospital stay (12.4 ± 12.0 vs. 9.3 ± 3.3, *p* = 0.214).

### 3.4. Incidence of Surgical Side Infections

SSIs needing surgical revision were observed in six cases (9.4%), with five SSIs occurring in the vancomycin group (12.82%) and one in the control group (4.0%) (*p* = 0.391). Of these six total SSIs, Gram-positive bacteria accounted for four SSIs (6.6%; Table 2). There was one infection with a mixed spectrum of Gram-positive and Gram-negative bacteria as well as fungi in the interventional group (n = 1, 16.7%). One fungoid infection was found in the interventional group. Specifics of each SSI case are given in Table 3. Univariable logistic regression revealed higher age at timepoint of surgery to be a significant predictor of SSI (OR 0.73, 95% confidence interval (CI) 0.52–0.95, *p* = 0.03). Furthermore, MMC as the underlying syndrome was a significant predictor of SSI (OR 15.7, 95%CI 2.22–139.76, *p* = 0.006), while patients with ICP or SMA showed no enhanced OR for SSI. A history of spinal surgery (OR, 7.5, 95%CI 1.04–151.19, *p* = 0.079) or undergoing revision surgery (OR 5.9, 95%CI 0.69–42.16, *p* = 0.075) reached no statistical significance for an association with higher Odds for SSI.

Bivariable logistic regression revealed no significant reduction in SSI incidence, including after adjusting for the possible confounders age, prior spine surgery, spinal revision surgery, or MMC, (Table 4).

### 3.5. Incidence of Possible Vancomycin-Associated Adverse Events

One case of anaphylaxis was reported in the control group (4.0%) and was caused by intravenous preoperative application of cefazolin. No adverse events linked to vancomycin application were reported (Table 3).

## 4. Discussion

In this study analyzing surgical treatment of pediatric patients with secondary scoliosis, we observed no significant differences in SSI rates between the group who were not receiving intraoperative, intrawound vancomycin application based on surgical risk assessment compared to those receiving it. Furthermore, we observed no adverse events due to the administration of up to 2 g of vancomycin at the surgical wound site in children. Regarding the pediatric patient cohort, despite the importance of infection prevention in this population, evidence on the effect of intraoperative vancomycin application is scarce.

Mallet et al. describes a significant decrease in SSI rates after applying vancomycin subfascially in combination with povidone-iodine in a population of children undergoing PSF for AIS. Roberto et al., as well as a recent systematic review, confirmed these findings for AIS and secondary scoliosis [11,22]. Thompson and the Growing Spine Study Group showed a significant reduction in infection rates between the vancomycin and control groups in a pediatric cohort with secondary early-onset scoliosis undergoing initial implantation of growing rods, distractions, and conversions to definitive instrumentation. The infection rates were comparable. On another note, no adverse events were recorded for the interventional group [15]. In contrast, a study by Garg et al. shows no effect of vancomycin on the occurrence of SSIs, but reports a higher incidence of SSI in the vancomycin group [14]. Our results agree with Garg’s work, as we observed a higher incidence of SSI in the vancomycin than in the interventional group (Vanco 12.8% vs. Ctrl 4.0%). This is especially interesting, as it suggests that the subjective assessment of the infection risk for the respective patient, which led to the indication to not use vancomycin, shows low infection rates when comparing to the literature [4,5]. Regardless of the aforementioned results, there might be a therapeutic effect of intrawound vancomycin application that is clinically relevant yet not graspable based on statistics. As there is no international consensus on when to use vancomycin, it is up to the surgeon to choose whether they use it. Experienced surgeons tend to use vancomycin in high-risk patients (higher ASA scores, intraoperative sterility concerns, steroid usage). In that case, one could hypothesize that vancomycin does reduce the SSI rate to comparable levels compared to our control group, which consisted of patients with a tendency towards a lower ASA score.

Furthermore, due to the high heterogeneity of published literature on this topic, no conclusive evidence is available so far. Sathish and Girinivasan have pointed this out in a recent review, concluding the heterogeneity among the studies makes it hard to properly evaluate the effect of vancomycin [23].

In contrast to the pediatric collective, the scientific discussion in adult spinal surgery regarding the effect of vancomycin in SSI prevention is much more mature. The effect of vancomycin has been the topic of many research groups, regardless of the etiology [24,25,26,27,28], with controversial results. A meta-analysis by Khan et al. showed a protective effect of the use of vancomycin powder in elective adult spinal surgery by analyzing retrospective studies and a randomized controlled trial. This showed an increased protective effect in instrumented procedures compared with non-instrumented procedures [24]. Agreeing with Khan’s result, a systematic review conducted by Ghobrial’s research group showed a significant reduction in SSI rates with the use of vancomycin (1.36%) compared with untreated controls (7.48%). Thus, the meta-analysis showed that the SSI rate in the untreated group was 7.48%, while the treated group had a rate of only 1.36%. Furthermore, the application was shown to be safe, with a rate of 0.3% adverse events (n = 23/6701) [17]. In contrast to the very straightforward results of the aforementioned research groups, a review paper by Vakayil et al., which involved a propensity-matched analysis, found that vancomycin did not lead to a significant reduction in SSI rates. As part of the unadjusted analysis, they observed an increased number of SSIs as well as an altered pathogen spectrum [26].

This introduces another important aspect of the scientific discussion about vancomycin installation: there is concern in the literature as well as in the context of our cohort’s results about exposing the surgical site to selection pressure leading to increased antimicrobial resistance, mixed microbial infections and isolated Gram-negative SSIs. In a pediatric cohort, Gans et al. found an increase in Enterobacter and MRSA after intraoperative vancomycin application [16]. In the adult population, Ghobrial et al. and Chotai et al. reported a higher proportion of Gram-negative infections in comparison to an untreated control group (28% Gram-negative infections in the treated group vs. 12.5% in the untreated group), while increased occurrence of vancomycin-resistant *Staphylococcus* species was not reported. Accordingly, the rate of *S. aureus* infections decreased with vancomycin application (32% in the Vanco group; 65% in the untreated group) [17,18]. Mirzahashi et al.’s results are in agreement with this finding, as they noted more SSIs being caused by Acitenobacter species as well as Pseudomonas. Also, polymicrobial infections tended to be higher in all of the abovementioned groups [18,29]. This is particularly concerning, as antimicrobial resistance is becoming a global threat to health, prompting the World Health Organization to call for a “Global Action Plan” [30,31]. Moreover, “difficult-to-treat” peri-implant infections may result in significantly worse long-term outcomes for affected patients compared to less resistant infections [32,33,34]. In adult spinal surgery, the emergence of an antimicrobial-resistant microbial spectrum has been already reported for patients requiring multiple revision surgeries [35], as well as for pediatric urinary tract infections [36]. Given the responsibilities towards the individual patient but also health as a common good in a global context, high awareness of the treating spinal surgeon and careful decision making seems advisable. This puts further emphasis on the need for a consensus of vancomycin application in both adult and pediatric spinal surgery.

Intraoperative vancomycin application has been linked to anaphylactic reactions, nephrotoxic reactions, acute renal vein failure, Stevens–Johnson or Red man syndrome [37]. Gans et al. identified undetectable serum vancomycin levels and negligible creatinine changes, ruling out the risk of vancomycin-induced nephropathy in children [16]. Thompson et al. and Armaghani et al. did not account for any cases of nephropathy [13,15]. Furthermore, Sweet et al. proved that while intra wound vancomycin application leads to supratherapeutic local levels, the systemic effect of intrawound vancomycin application is neglectable in adults [38]. Regarding more recent works, incidental case reports of interstitial nephritis after implantation of vancomycin-loaded bone cement in knee and hip arthroplasty have been published [39]. Ghobrial’s study group performed an extensive meta-analysis and reported 23 adverse events in 6.701 patients (0.3%) with nephropathy occurring in 1 case, ototoxicity resulting in transient hearing loss in 2 patients with supratherapeutic vancomycin levels through systemic absorption, and mostly postoperative culture-negative seroma formation in 19 patients, further undermining the safety of application [17]. These findings are in line with our results, as we did not find any acute, major vancomycin-related adverse events after the application of 2 g of vancomycin.

Nevertheless, it is important to consider that subtle or long-term adverse effects were not examined. Given the concerns about nephrotoxicity and antibiotic resistance [40], a more rigorous monitoring and reporting framework for subtle and long-term adverse events would have been beneficial and is encouraged for further studies.

It is therefore important to underscore that vancomycin application is only one of the tools used for SSI prevention. Our study protocol also involved a thorough disinfection of the skin and the usage of an incision foil, as well as preoperative decolonization in patients with known MRSA. Furthermore, it emphasized a straightforward surgical process to reduce surgical time and personnel rotation during surgery. These measures have been shown to reduce the risk of SSI in pediatric scoliosis surgery as well as orthopedic surgery, respectively [41,42]. Notably, in the lower-risk group without interwound vancomycin, we observed a comparably low SSI rate of 4%, indicating that the absence of vancomycin did not lead to a high SSI rate, reinforcing that vancomycin application is not always necessary.

Limitations: Our study has several limitations that must be acknowledged. First, the retrospective design limits our ability to establish causality and introduces potential bias due to reliance on medical records.

The study size of 64 patients in combination with the comparable low incidence of SSI for secondary scoliosis surgery limits the statistical power of this study and increases the risk of type II errors, missing clinically significant differences. Although we observed similar SSI rates between the vancomycin and control groups, the limited sample size prevents us from ruling out a potentially meaningful effect. Although presentation with delayed SSI usually triggers referral to our center for specialized treatment, we cannot rule out missed late SSI cases, or late non-surgical complications due to the retrospective design. The event count limited the number of possible confounders which could be adjusted for in logarithmic regression to a bivariate analysis and this should be considered when interpreting our findings. Furthermore, we have included patients with a history of spinal surgery. This can be an independent risk factor for development of SSI [43]. We have decided to include these patients for two reasons: First, our subgroup analysis for vancomycin application in children undergoing definitive PSF as their first surgery has not influenced our results. Second, we believe that we are better able to represent the heterogeneity in children with secondary scoliosis. As the process of determining which patients received vancomycin based on perceived risk could introduce bias, careful interpretation is warranted. While the strength of our single-center single-surgeon series lies in the uniformity of surgical indications and performance of one surgeon, this limits the possibility of generalizing the results of our study. Therefore, to sustainably determine the effectiveness of vancomycin application in pediatric patients with secondary scoliosis, a larger prospective, multi-center study is needed to provide more robust data, better evidence, and limit bias, ideally in a randomized design. The focus of future research should be based on the incidence of SSI, the changes in pathogen spectrum in the cases of SSI, as well as the incidence of adverse events. Future research should aim to establish an international consensus of SSI as well as vancomycin-related adverse events in children with secondary scoliosis based on both clinical examination and changes in pre- to postoperative blood testing.

## 5. Conclusions

In conclusion, in our patient cohort of children with secondary scoliosis who underwent PSF or implantation of a growing rod system, we observed comparable incidences of SSI in patients who did not receive intraoperative vancomycin compared to those who did, which was also the case after adjusting for possible confounders. While we observed no adverse events of intraoperative vancomycin application in children, we did observe an altered pathogen spectrum in those who received intrawound vancomycin application. Patients with MMC, older age, or further risk factors for vancomycin-sensible SSI may benefit from vancomycin; while low-risk cases might not need it, those who did not receive intrawound vancomycin showed a low SSI rate of 4%. Therefore, this study provides a contribution to the scientific discussion, advocating for more elaborate vancomycin application, as the risks of pathogen spectrum alteration may exceed the benefits.

## Figures and Tables

**Table 1:** Study cohort characteristics.

	Total N = 64	Vanco N = 39	No Vanco N = 25	*p* ^1^
Age (years, mean, SD)	12.6 ± 3.0	12.6 ± 2.7	12.7 ± 3.4	0.633
Sex				0.447
female	35 (54.7%)	23 (59.0%)	12 (48%)	
male	29 (45.3%)	16 (41.0%)	13 (52%)	
Body weight (kg, SD)	36.5 ± 14.2	35.8 ± 13.2	37.7 ± 15.7	0.597
Height (m, SD)	1.42 ± 0.18	1.41 ± 0.18	1.43 ± 0.19	0.424
BMI (SD)	17.8 ± 4.8	17.7 ± 4.7	17.8 ± 5.0	0.960
Syndrome: n (%)				0.161
ICP	13 (20.3%)	2 (8.0%)	11 (28.2%)	
MMC	8 (12.5%)	3 (12.0%)	5 (12.8%)	
SMA	6 (9.4%)	2 (8.0%)	4 (10.3%)	
Muscle dystrophies	9 (14.1%)	5 (20.0%)	4 (10.3%)	
specific syndrome	8 (12.5%)	2 (8.0%)	6 (15.4%)	
unspecific syndrome	5 (7.8%)	1 (4.0%)	4 (10.3%)	
Neurofibromatosis	3 (4.7%)	2 (8.0%)	1 (2.6%)	
Others	12 (18.8%)	8 (32.0%)	4 (10.3%)	
ASA				
I	0	0	0	0.114
II	17 (26.6%)	8 (20.5%)	9 (36%)	
III	45 (70.3%)	29 (74.4%)	16 (64%)	
IV	2 (3.1%)	2 (5.1%)	0	
Previous spine surgery				
(mean, SD)	1.3 ± 2.7	1.1 ± 1.8	1.7 ± 3.7	
0	39 (60.9%)	24 (61.5%)	15 (60%)	
1	10 (15.6%)	5 (12.8%)	5 (20%)	0.941
2	4(6.3%)	3 (7.7%)	1 (4%)	
>2	11 (17.2%)	7 (17.9%)	4 (16%)	

^1^ Two-tailed *t*-test, Mann-Whitney U test or Fisher’s exact test as appropriate. kg = kilogram; SD = standard deviation; ASA = American Society of Anesthesiology Classification, ICP = infantile cerebral palsy, MMC = myelomeningocele, SMA = spinal muscle dystrophy.

**Table 2:** Length of stay and complications.

	Total N = 64	Vanco N = 39	No Vanco N = 25	*p* ^1^
ICU stay				0.497
Mean (days, SD)	2.5 ± 3.1	2.4 ± 3.1	2.7 ± 3.1	
No	16 (25.0%)	11 (28.2%)	5 (20.0%)	
1 d	16 (25.0%)	11 (28.2%)	5 (20.0%)	
2 d	11 (17.2%)	7 (17.9%)	4 (16.0%)	
3–5 d	13 (20.3%)	6 (15.4%)	6 (24.0%)	
>5 d	8 (12.5%)	4 (10.3%)	4 (16.0%)	
Hospital stay				0.214
Mean (SD)	10.5 ± 8.1	9.3 ± 3.3	12.4 ± 12.0	
<3 d	0	0	0	
3–7 d	14 (21.9%)	10 (25.6%)	4 (16.0%)	
7–14 d	45 (70.3%)	26 (66.7%)	19 (76.0%)	
>14 d	5 (7.8%)	3 (7.7%)	2 (8.0%)	
Complications				
Neurological Deterioration	1(1.6%)	1 (2.6%)	0	n.s.
Cardiac Arrest	0	0	0	n.s.
SSI with wound revision	6 (9.4%)	5 (14.7%)	1 (4.0%)	0.391
SIRS	9 (5.8%)	4 (10.3%)	5 (20.0%)	0.296
further revision surgery	2 (3.13%)	2 (5.13%)	0	0.516
respiratory insufficiency	28 (43.8%)	16 (41.0%)	12 (48%)	0.615
Anaphylaxis	0	0	1 (4.0%)	n.s.

^1^ Chi-square test, Mann-Whitney U test, Fisher’s exact test as appropriate. d = days; n.s. = not significant; SD = standard deviation; SIRS = Systemic inflammatory response syndrome; SSI = Surgical Site Infection.

**Table 3:** Specifics of SSI cases.

	Underlying Syndrome	Pathogen	Post-Op. i.v. Antibiotics	Post-Op p.o. Antibiotics
**Control**				
Case 1	MMC	*S. epidermidis*	Unacid 3 g i.v.; Daptomycin 500 mg i.v.	Ciprofloxacin 500 mg + Rifampicin 450 mg (12 weeks)
**Vancomycin**				
Case 1	MMC	*P. aeruginosa* *E. coli* *P. mirabilis*	Tazobac 4.5 g; Vancomycin 500 mg	AmoxiClav 1 g (4 weeks)
Case 2	MMC	*S. epidermidis*	Tazobac 4.5 g; Fofomycin/Daptomycin	Ciprofoloxacin 500 mg; Rifampicin 300 mg (12 Weeks)
Case 3	SMA	*P. acnes*	Unacid 3 g	Ciprofloxacin 500 mg; Rifampicin 300 mg (12 Weeks)
Case 4	MMC	*C. glabrata*	Voriconazoli.v.	Voriconazol 200 mg (12 Weeks)
Case 5	ICP	*S. aureus*	Cefazolin 1 g i.v. plus Fosfomycin 3 g	Cotrimoxazol 960 mg; Rifampicin 300 mg (12 Weeks)

ICP = infantile cerebral palsy; MMC = meningomyelocele; SMA = spinal muscular atrophy.

**Table 4:** Univariable and bivariable logistic regression analyzing potential risk and protective factors and their association with the occurrence of SSI in need of surgical revision.

	SSI		
Predictors	OR ^1,2^	CI	*p*
Age	0.73	0.52–0.95	**0.030**
Sex [male]	0.23	0.01–1.65	0.198
ASA	0.24	0.03–1.46	0.131
MMC [1]	15.75	2.22–139.76	**0.006**
ICP [1]	0.98	0.05–7.32	0.988
Prior spinal surgery [1]	7.5	1.04–151.19	0.079
Revision surgery [1]	5.9	0.69–42.16	0.075
Vanco [1]	3.67	0.51–73.70	0.256
(unadjusted)			
Vanco [1] adjusted for	4.73	0.54–114.82	0.219
Age	0.69	0.47–0.93	**0.031**
Vanco [1] adjusted for	4.73	0.60–102.60	0.195
revision surgery [1]	7.56	0.82–64.13	0.055
Vanco [1] adjusted for	3.98	0.53–82.21	0.237
prior spinal surgery [1]	7.9	1.07–161.34	0.074
Vanco [1] adjusted for	3.16	0.37–68.28	0.341
MMC [1]	14.63	2.00–133.48	**0.009**

^1^ Univariable logistic regression model. ^2^ Bivariable logistic regression model adjusting for the displayed confounder. ASA = American Society of Anesthesiology Classification, ICP = infantile cerebral palsy, MMC = myelomeningocele.

## Data Availability

The datasets generated and analyzed during the current study are available from the corresponding author on reasonable request.
